# Chemical Composition and Biological Activity of Essential Oil and Extract from the Seeds of *Tropaeolum majus* L. var. *altum*

**DOI:** 10.17113/ftb.60.04.22.7667

**Published:** 2022-12

**Authors:** Ivana Vrca, Dina Ramić, Željana Fredotović, Sonja Smole Možina, Ivica Blažević, Tea Bilušić

**Affiliations:** 1 Department of Food Technology and Biotechnology, Faculty of Chemistry and Technology, University of Split, Ruđera Boškovića 35, 21000 Split, Croatia; 2 Department of Food Science and Technology, Biotechnical Faculty, University of Ljubljana, Jamnikarjeva ulica 101, 1000 Ljubljana, Slovenia; 3 Department of Biology, Faculty of Science, University of Split, Ruđera Boškovića 33, 21000 Split, Croatia; 4 Department of Organic Chemistry, Faculty of Chemistry and Technology, University of Split, Ruđera Boškovića 35, 21000 Split, Croatia

**Keywords:** *Tropaeolum majus* L., benzyl isothiocyanate, benzyl cyanide, antiproliferative activity, antimicrobial activity

## Abstract

**Research background:**

Plant *Tropaeolum majus* L. (garden nasturtium) belongs to the family Tropaeolaceae and contains benzyl glucosinolate. The breakdown product of benzyl glucosinolate, benzyl isothiocyanate (BITC), exhibits various biological activities such as antiproliferative, antibacterial and antiinflammatory. In order to optimize the content of biologically active volatile compounds in plant extract and essential oil, the use of appropriate extraction technique has a crucial role.

**Experimental approach:**

The current study investigates the effect of two modern extraction methods, microwave-assisted distillation (MAD) and microwave hydrodiffusion and gravity (MHG), on the chemical composition of volatile components present in the essential oil and extract of garden nasturtium (*T. majus* L. *var. altum*) seeds. Investigation of the biological activity of samples (essential oil, extract and pure compounds) was focused on the antiproliferative effect against different cancer cell lines: cervical cancer cell line (HeLa), human colon cancer cell line (HCT116) and human osteosarcoma cell line (U2OS), and the antibacterial activity which was evaluated against the growth and adhesion of *Staphylococcus aureus* and *Escherichia coli* to polystyrene surface.

**Results and conclusions:**

Essential oil and extract of garden nasturtium (*T. majus*) seeds were isolated by two extraction techniques: MAD and MHG. BITC and benzyl cyanide (BCN) present in the extract were identified by gas chromatography-mass spectrometry. Essential oil of *T. majus* showed higher antiproliferative activity (IC_50_<5 µg/mL) than *T. majus* extract (IC_50_<27 µg/mL) against three cancer cell lines: HeLa, HCT116 and U2OS. BITC showed much higher inhibitory effect on all tested cells than BCN. The essential oil and extract of *T. majus* showed strong antimicrobial activity against *S. aureus* and *E. coli*.

**Novelty and scientific contribution:**

This work represents the first comparative report on the antiproliferative activity of the essential oil and extract of *T. majus* seeds, BITC and BCN against HeLa, HCT116 and U2OS cells as well as their antimicrobial activity against *S. aureus* and *E. coli*. This study demonstrates that the essential oil of *T. majus* seeds exhibits stronger antiproliferative and antimicrobial activity than the plant extract.

## INTRODUCTION

Plant *Tropaeolum majus* L. (garden nasturtium) belongs to the family Tropaeolaceae, and is known for its ornamental and medicinal uses (*1*). Thanks to its therapeutic properties, it is also used in traditional medicine for the treatment of various diseases: externally as a disinfectant, for treatment of burns and diaper rash, and internally as a good remedy for the treatment of cancer, bronchitis, tuberculosis and asthma (*2*). It is also known for its antibacterial, antifungal and antiviral activities, and is therefore used as pharmacological agent for the treatment of acute sinusitis and urinary tract infections (*3*).

The leaves and seeds of *T. majus* contain fatty acids, flavonoids, tetracyclic triterpenes of cucurbitin and glucosinolates (GSLs) - benzyl glucosinolate and sinalbin (*4*). According to Bloem *et al*. (*5*), *T. majus* contains only one GSL: benzyl GSL (glucotropaeolin).

GSLs are specialized plant metabolites found in the botanical order Brassicales, in which the Brassicaceae represents the largest family (*6*). GSLs are present in all parts of *T. majus*, especially in the leaves, flowers and seeds (*7*). Intact GSLs are not biologically active compared to their breakdown products, especially isothiocyanates (ITCs), which are reported to be very active against a wide range of organisms (*7*), including microrganisms such as bacteria. GSLs are hydrolyzed by enzyme myrosinase contributing to the formation of various degradation products (ITCs, thiocyanates and nitriles) that depend on physiological conditions such as pH and the presence of certain cofactors like epithiospecifier protein (ESP) (*8*, *9*). ITCs are formed at neutral pH, while at acidic pH nitriles are dominant products (*10*). Solvent-free extraction methods such as microwave-assisted distillation (MAD), and microwave hydrodifussion and gravity (MHG) are excellent replacements for conventional extraction methods in order to obtain isolates rich in volatile compounds (*9*). These new extraction methods are much faster, easier, enviromentally friendly and they enable the extraction of biologically active compounds with reduced energy (*9*). ITCs can potentially be used to prevent various cancers such as lung, liver, breast and colon cancers (*11*). Due to their ability to cause growth arrest and cell death selectively in cancer cells, cancer prevention with dietary ITCs is ready for clinical translational research (*12*). *T. majus* has been reported to have anticancer activity (*13*, *14*), which can be explained by the presence of benzyl isothiocyanate (BITC) – a degradation product of benzyl GSL (*1*, *15*–*17*). BITC has shown excellent antiproliferative activity against human colon cancer cell line HT-29 (*17*). According to Xie *et al*. (*18*), the BITC treatment leads to suppression of tumour growth, inhibition of cellular proliferation and increased apoptosis in the breast tumours. However, the data on the anticancer activity of *T. majus* volatile sulphur compounds called essential oil and extract obtained by microwave technique is lacking. Moreover, ITCs are volatile compounds that have inhibitory effects even at low concentrations on various pathogenic microorganisms, making them desirable antimicrobial agents (*19*). One of the important biological properties of *T. majus* is antimicrobial activity, and is attributed to the presence of BITC (*20*). Antimicrobial activity of BITC has been investigated (*21*–*23*), while the research on the antimicrobial activity of *T. majus* essential oil and extract is scarce. Bazylko *et al*. (*20*) reported that aqueous and hydroethanolic extracts obtained from air-dried and freeze-dried *T. majus* did not have antimicrobial activity against *Staphylococcus aureus*, *Bacillus subtilis*, *Micrococcus luteus*, *Escherichia coli*, *Pseudomonas aeruginosa* and *Bordetella bronchiseptica*. The main explanation is probably a low content of BITC in the extracts. There are also some reports that aromatic ITCs, such as BITC, have stronger antibacterial activity than the aliphatic ones against plant pathogenic bacteria, foodborne pathogens, spoilage bacteria and methicillin-resistant *S. aureus* (*21*). Dias *et al*. (*23*) reported that among all tested ITCs against 15 isolates of methicillin-resistant *S. aureus*, the BITC was the most efficient. Bacterial microorganisms can adhere to the surface of extracellular polymeric substances, forming a biofilm (*24*). Bacteria in the planktonic state are less resistant to usual antibacterial compounds and substances than the bacteria in biofilms (*24*), which is why it is important to identify new natural components with high antibacterial activity and the ability to form biofilm.

The aims of this study are to obtain the essential oil and extract of *T. majus* with two modern extraction techniques: MAD and MHG, to determine volatile components in the prepared samples by gas chromatography-mass spectrometry (GC-MS), to examine antiproliferative activity of the essential oil, extract and pure compounds benzyl isothiocyanate (BITC) and benzyl cyanide (BCN)) against cervical cancer cell line (HeLa), human colon cancer cell line (HCT116) and human osteosarcoma cell line (U2OS), and to determine the antibacterial activity of the prepared samples and pure compounds against the growth of *S. aureus* and *E. coli* and their adhesion to the polystyrene surface.

## MATERIALS AND METHODS

### Plant material and reagents

The seeds of *Tropaeolum majus* L. var. *altum* (garden nasturtium) were purchased from Marcon d.o.o. (Novi Marof, Croatia). Before the extraction of volatile compounds, the seeds were milled to a fine powder using a coffee grinding machine (Gorenje, Velenje, Slovenia). Afterwards, the ground seeds were soaked in distilled water directly before microwave-assisted distillation (MAD), and approx. 1 h before microwave hydrodiffusion and gravity (MHG) extraction. BITC, BCN and benzaldehyde were commercially purchased from Sigma-Aldrich, Merck KgaA (Darmstadt, Germany). All other used chemicals and reagents were of analytical grade.

### Microwave-assisted isolation

The essential oil and extract of *T. majus* were isolated by MAD and MHG extraction techniques, respectively, using an ETHOS X device (Milestone, Italy) and applying microwave power of 500 W as desribed by Vrca *et al*. (*1*, *9*). The essential oil was extracted from the seeds (50 g). The microwave oven temperature was approx. 98 °C, and the extraction time was set to 30 min. After the MAD, the *T. majus* essential oil, collected in the pentane trap, was dried with anhydrous sodium sulfate to remove any residual water. Extract of *T. majus* seeds (50 g) was isolated by setting the device to flavour option and time to 15 min. The extract was collected in a glass beaker at the bottom of the apparatus, filtered and extracted with dichloromethane from water extract. The volatile isolates were stored in vials at -20 °C until further analysis.

### GC-MS analysis

Gas chromatographic system used for quantification contained gas chromatograph, model 8890 GC, equipped with an automatic liquid injector, model 7693A, and tandem mass spectrometer (MS/MS), model 7000D GC/TQ (Agilent Technologies Inc., Santa Clara, CA, USA). The volatile isolates were analyzed on a non-polar HP-5MS UI column (length 30 m, inner diameter 0.25 mm and stationary phase layer thickness 0.25 μm, Agilent Technologies Inc.). The column temperature program was adjusted at 60 °C for the first 3 min and then heated to 246 °C at 10 °C/min, and maintained for 3 min isothermally. Helium was the carrier gas and the flow rate was 1 mL/min. The inlet temperature was 250 °C and the volume of the injected sample was 1 μL. Additional conditions were: ionization energy 70 eV, transfer line temperature 280 °C, ion source temperature 230 °C, the temperature of the quadrupoles was set at 150 °C, while the flow of nitrogen through the collision cell was 1.5 mL/min. The individual peaks were identified (relative to C_8_-C_40_ *n*-alkanes for HP-5MS UI column, Agilent Technologies Inc) by comparison of their retention indices and mass spectra with those of authentic samples, as well as by computer matching against the Wiley 9N08 MS (*25*) and NIST17 mass spectral databases (*26*).

### Antiproliferative activity

The antiproliferative activity of *T. majus* essential oil and extract, as well as pure compounds BITC and BCN was determined on HeLa, HCT116 and U2OS cancer cell lines kindly given by Prof. Janoš Terzić (School of Medicine, University of Split) as described by Fredotović *et al*. (*27*). The antiproliferative activity was determined using the tetrazolium compound MTS-based CellTiter 96® aqueous assay (Promega, Madison, WI, USA). Cells were grown in a CO_2_ incubator at 37 °C and 5% CO_2_ until they reached 80% confluency. Afterwards, the cells were counted using the automatic handheld cell counter (Merck, Darmstadt, Germany), and 0.5·10^4^ cell/well were seeded in 96-well plates and treated with serially diluted extracts. Cells were further grown for 48 h, after which 20 µL of MTS tetrazolium reagent (Promega) were added to each well. The absorbance was measured at 490 nm using a 96-well plate reader (model EL808; Bio-Tek, Winooski, VT, USA) after 3 h of incubation at 37 °C and 5% CO_2_. Solvent control was measured and incorporated into the obtained results. Experiments were carried out in four replicates for each concentration and IC_50_ values were calculated from three independent experiments using GraFit 6 data analysis software (*28*). For statistical analyses, the two-way ANOVA and Sidak's multiple comparisons tests were used and statistically significant differences were at p<0.0001.

### Bacterial strains and growth conditions

Gram-positive *Staphylococcous aureus* ATCC 25923 and Gram-negative *Escherichia coli* ATCC 11229, which are part of the collection of microorganisms of the Laboratory for Food Microbiology of the Department of Food Science and Technology, Biotechnical Faculty, University of Ljubljana, were used for antibacterial testing. The bacteria were stored at -80 °C in tryptic soy broth (Biolife, Milan, Italy) together with 15% glycerol (Kemika, Zagreb, Croatia), revitalised on Mueller-Hinton (MH) agar (bioMérieux, Marcy-l'Étoile, France) and incubated aerobically at 37 °C for 24 h. Standardised inocula with 10^5^ colony-forming units (CFU) per mL were prepared in MH broth (Oxoid, Hampshire, UK) for antibacterial assays.

### Antibacterial susceptibility

Microdilution method was used to determine the minimal inhibitory concentrations (MICs). Briefly, the essential oil, extract and pure compounds (BITC and BCN) were dissolved and diluted in absolute ethanol (Merck). Twofold serial dilutions of the essential oil, extract and pure compounds were made in a 96-well microtiter plate to achieve concentrations from 2 to 0.06 mg/mL with the final volume 50 µL. A volume of 50 µL of the prepared inoculum (10^5^ CFU/mL) was added to each well and mixed. A volume of 10 µL 2-*p*-iodophenyl-3-*p*-nitrophenyl-5-tetrazolium chloride (INT; Sigma-Aldrich, Merck, St. Louis, MO, USA) was added after incubation and it was used as an indicator for bacterial metabolic activity (*29*). The lowest concentration at which bacterial growth was not detected as a reduction of INT to red formazan was MIC. To avoid the inhibitory effect on the growth of the selected bacteria, the ethanol volume fraction in the assay never exceeded 1%. To determine minimal bactericidal concentrations (MBCs), 5 µL of cultures, collected from each well of 96-well microtiter plate, were inoculated on MH agar and then incubated aerobically for 24 h at 37 °C. The MBC was determined as the concentration at which bacteria did not grow.

### Bacterial growth kinetics

The essential oil, extract and pure compounds, BITC and BCN, were added to 5 mL of growth medium to give final concentrations from 0.25 to 0.031 mg/mL for *S. aureus* ATCC 25923 and from 1 to 0.125 mg/mL for *E. coli* ATCC 11229. Furthermore, BITC and BCN were tested together at ratios 1:2 and 1:1 against *S. aureus* ATCC 25923 and *E. coli* ATCC 11229, regarding the results obtained for MIC. The cultures of *S. aureus* or *E. coli* without the addition of plant preparations were used as a positive control. For negative control, MH broth with or without the addition of plant preparations in different concentrations was used and after measurements it was deducted from the obtained results. Inocula were prepared as described above. Volumes of 100 µL of the prepared cultures and negative controls, with or without the addition of plant preparations, were added to 96-well microtiter plates (Nunc 266120 polystyrene plates; Nunc, Roskilde, Denmark). The absorbance was measured at 600 nm using multiskan reader (Thermo Fisher Scientific, Waltham, MA, USA) every 30 min for 24 h at 37 °C to obtain growth curves.

### Antiadhesion assay

The adhesion of *S. aureus* ATCC 25923 and *E. coli* ATCC 11229 was analysed in treatments with essential oil, extract, BITC and BCN. Inocula were prepared as described above and treated with the essential oil, extract, BITC and BCN at MIC, ½ MIC and ¼ MIC. The treated inocula (200 µL) were then transferred to 96-well polystyrene microtitre plates (Nunc 266120 polystyrene plates; Nunc) and incubated aerobically at 37 °C for 24 h. To remove the non-adherent cells, each well in the microtiter plate was rinsed three times with phosphate-buffered saline (PBS; Oxoid), then 200 µL of PBS were added to each well and sonicated for 10 min (28 kHz, 300 W; Iskra Pio, Šentjernej, Slovenia). The adhesion of the cells was expressed in CFU/mL as previously described by Šikić Pogačar *et al*. (*30*). The untreated culture was used as the negative control.

### Statistical analysis

Experiments were carried out in triplicate as three or more independent experiments. The data were analysed with Origin 2018 (*31*) and expressed as mean value±standard deviation (S.D.). IBM SPSS Statistics 23 (*32*) was used to perform statistical analysis. The Kolmogorov-Smirnov test of normality was used to determine the distribution of data and statistical significances were determined using *t-*test for two independent mean values. Data were significant at p<0.05.

## RESULTS AND DISCUSSION

### Chemical composition of the essential oil and extract of T. majus seeds

The volatile components from benzyl glucosinolate (GSL) from garden nasturtium (*Tropaeolum majus*) seeds obtained after microwave hydrodiffusion and gravity (MHG) were identified by GC-MS. The analysis showed that the main volatile compounds in the extract of *T. majus* were benzyl cyanide (BCN) and benzyl isothiocyanate (BITC) (37.00 and 54.35%, respectively) (Table 1). Vrca *et al*. (*1*) reported that BITC was the main component in the essential oil (97.81%), while BCN was present in low amount (0.80%) after 30 min of treatment and application of 500 W microwave power using microwave-assisted distillation (MAD). The higher amount of BCN in the extract after MHG can be explained by the presence of epithiospecifier protein (ESP). The interaction of the ESP with the enzyme myrosinase diverts the reaction towards the production of epithionitriles or nitriles, depending on the glucosinolate structure (*33*). Given that ESP is thermally sensitive, it is known that its activity decreases significantly at 50 °C or higher (*34*), which is the main reason for the low presence of BCN in the essential oil of *T. majus*. On the other hand, soaking for 1 h in water before MHG technique enabled the formation of BCN, which explains its high percentage in the the seed extract.

**Table 1:** Chemical composition of volatile compounds in *Tropaeolum majus* extract after microwave-assisted isolation

Volatile compound		RI	*φ*/%		
MHG		MAD*
Benzaldehyde		962	4.42		-
BCN	1144			37.00	0.80 97.81
BITC	1369			54.35	
Total			95.77		98.61

*Vrca *et al*. (*1*); RI=retention, MHG=microwave hydrodiffusion and gravity, MAD=microwave-assisted distillation, BCN=benzyl cyanide, BITC=benzyl isothiocyanate

According to Wielanek *et al*. (*35*), after *in vitro* hydrolysis of benzyl GSL in hairy root cultures of *T. maju*s, degradation products were BITC, BCN and benzyl thiocyanate (BTC). We did not detect BTC in the analyzed samples. The enzymatic formation of organic thiocyanates is believed to require thiocyanate-forming protein (TFP). However, recent report by Todorovska-Rašić and Radulović (*36*) suggests they could be formed *via* metabolic routes that do not involve TFP.

### Antiproliferative activity of T. majus essential oil and extract

**Fig. 1:**
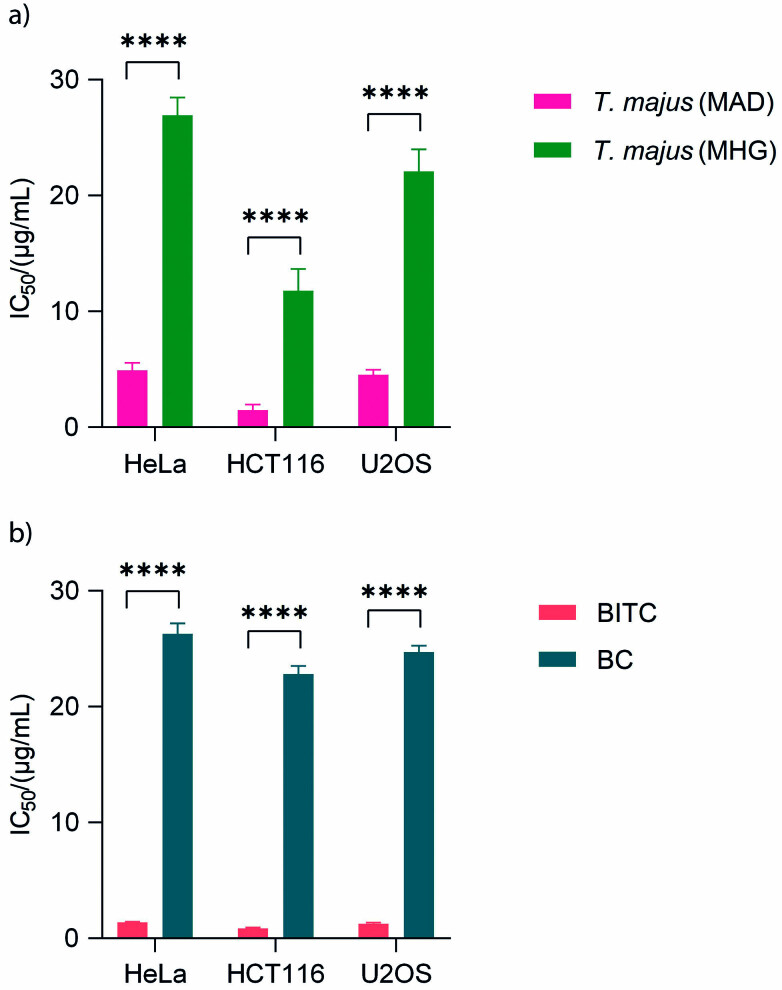
Antiproliferative activity of *Tropaeolum majus*: a) essential oil after microwave-assisted distillation (MAD) and extract after microwave hydrodiffusion and gravity (MHG), and b) antiproliferative activity of benzyl isothiocyanate (BITC) and benzyl cyanide (BCN) on HeLa, HCT116 and U2OS determined by MTS cell proliferation assay. The results are expressed as mean value of three independent experiments with S.D. values (presented as error bars). For statistical analyses, the two-way ANOVA and Sidak's multiple comparisons tests were used. ****Statistically significant differences at p<0.0001

To the best of our knowledge, the antiproliferative activity of the essential oil and extract of *T. majus*, as well as of pure compounds BITC and BCN was determined for the first time on HeLa, HCT116 and U2OS cancer cell lines using MTS cell proliferation assay (Fig. 1). Cancer cells were treated for 48 h, and the results were expressed as IC_50_ values (50% cell growth inhibitory concentrations). The essential oil of *T. majus* had better effect on all three cancer cell lines: HeLa, HCT116 and U2OS (IC_50_=4.91, 1.49 and 4.53 µg/mL respectively) than the plant extract (IC_50_=26.93, 11.79 and 22.09 µg/mL respectively). BITC showed much higher inhibitory effect on all tested cells (IC_50=_1.39, 0.85 and 1.27 µg/mL) than BCN (IC_50_=26.29, 22.82 and 24.7 µg/mL).

BITC has been reported to exhibit antitumour activities in various types of carcinoma (*11*). At concentrations of BITC>10 μM (1.49 µg/mL), the survival rate of 4T1-Luc murine mammary carcinoma cells was <50% using MTT assay (*11*). Previous studies on *in vitro* cell culture revealed that BITC showed anticancer activity by inducing apoptosis and G2/M cell cycle arrest in different cancer cells (breast cancer, lung cancer, pancreatic cancer and leukemia cells) at concentrations between 2 and 100 µmol/L (0.30–14.92 µg/mL) (*15*). These results are consistent with ours on HeLa, HCT116 and U2OS cancer cell lines, where IC_50_<2 µg/mL was for pure compound BITC, and IC_50_<5 µg/mL for the plant essential oil. Results of MTT assay showed that BITC (5–20 µmol/L or 0.75–2.98 µg/mL) reduced the number of viable TRAMP-C2 prostate cancer cells and DU145 human prostate cancer cells (5–10 µmol/L or 0.75–1.49 µg/mL) in a dose-dependent manner (*15*). According to Veeranki *et al*. (*16*), BITC inhibits osteosarcoma, which is consistent with our results obtained against human osteosarcoma cell line (U2OS). Huong *et al*. (*37*) also demonstrated that ITCs have high antiproliferative activity against human cervical cancer cell lines (HEp-2 and KB) using the MTS assay. They reported a significant decrease in cell viability by 2-phenylethyl ITC (PEITC), which makes it a potent growth inhibitor of cervical cancer cells.

Nastruzzi *et al*. (*38*) reported that ITC from benzyl glucosinolate appears to be the most active compound of all tested degradation products from various GSLs against human K562 erythroleukemic cell line, with a 50% cell growth inhibition at concentrations 1–6 μM (0.15–0.90 µg/mL). Nitrile from benzyl GSL had lower antiproliferative activity against human K562 erythroleukemic cell line than BITC (*38*), which is consistent with our results where IC_50_ for BCN was <27 µg/mL. According to the criteria used to categorize the activity against the tested cell lines that were based on IC_50_ values (*39*), our results suggest highly active antiproliferative activity of *T. majus* essential oil and pure compound BITC on all three tested cancer cell lines. The plant extract also showed strong antiproliferative activity on HCT116 cancer cell line, and moderate antiproliferative activity on other tested cancer cell lines (HeLa and U2OS). BCN showed moderate antiproliferative activity on all tested cancer cell lines. BITC has anticancer activity at concentrations (*in vitro*) or doses (*in vivo*) that are not toxic to normal tissues (*40*). The results presented in our study show that the degradation product of benzyl GSL, in our case BITC, has high biological activity and it could be used as a new anticancer agent for the prevention of various types of cancer.

### Antibacterial activity

Table 2 shows the results of antibacterial activity of pure compounds (BITC and BCN), essential oil after MAD and the extract after MHG. According to the obtained results, *S. aureus* was more sensitive to the above-mentioned samples than *E. coli*. Essential oil and extract had the same effect against *S. aureus* with MIC=0.0625 mg/mL and MBC=0.125 mg/mL, while the effect of BITC and BCN was twice weaker. The essential oil and extract were also effective against *E. coli,* with MIC=0.25 mg/mL and MBC=0.5 mg/mL. BITC had MIC=0.5 mg/mL and MBC=1 mg/mL against *E. coli*, while BCN had twice weaker effect.

**Table 2:** Minimal inhibitory (MIC) and bactericidal concentrations (MBC) against *S. aureus* ATCC 25923 and *E. coli* ATCC 11229 of pure compounds (BITC and BCN), plant essential oil after MAD and plant extract after MHG

Pure compound and plant preparation	*γ*/(mg/mL)			
*S. aureus* ATCC 25923		*E. coli* ATCC 11229	
MIC	MBC	MIC	MBC
BITC	0.125	0.25	0.5	1.0
BCN	0.125	0.25	1.0	2.0
Essential oil after MAD	0.0625	0.125	0.25	0.5
Extract after MHG	0.0625	0.125	0.25	0.5

BITC=benzyl isothiocyanate, BCN=benzyl cyanide, MAD=microwave-assisted distillation, MHG=microwave hydrodiffusion and gravity

ITCs are considered to be the most interesting compounds that are obtained by degradation of glucosinolates and are known as significant growth inhibitors of Gram-negative and Gram-positive pathogenic bacteria (*24*). Among all tested ITCs (allyl isothiocyanate, BITC and PEITC) against several isolates of methicillin-resistant *S. aureus*, the BITC was the most effective with a MIC varying from 2.9 to 110 μg/mL (*23*), which is in line with our results. The higher efficiency of BITC against 15 isolates of methicillin-resistant *S. aureus* than of some other ITCs (PEITC and allyl isothiocyanate) can be attributed to the aromatic ring and short carbon chain (*23*). According to Ko *et al*. (*21*), MIC and MBC of BITC were fourfold higher than our results (MIC=(0.500±0.000) mg/mL, and MBC˃1000 mg/mL). Kaiser *et al*. (*24*) reported that mean MIC for BITC against all tested *Pseudomonas aeruginosa* isolates was (2145±249) µg/mL. Consequently, degradation products of benzyl GSL (especially BITC) are promising alternative antibacterial agents.

### Effect of volatile compounds from T. majus seeds on bacterial growth

**Fig. 2:**
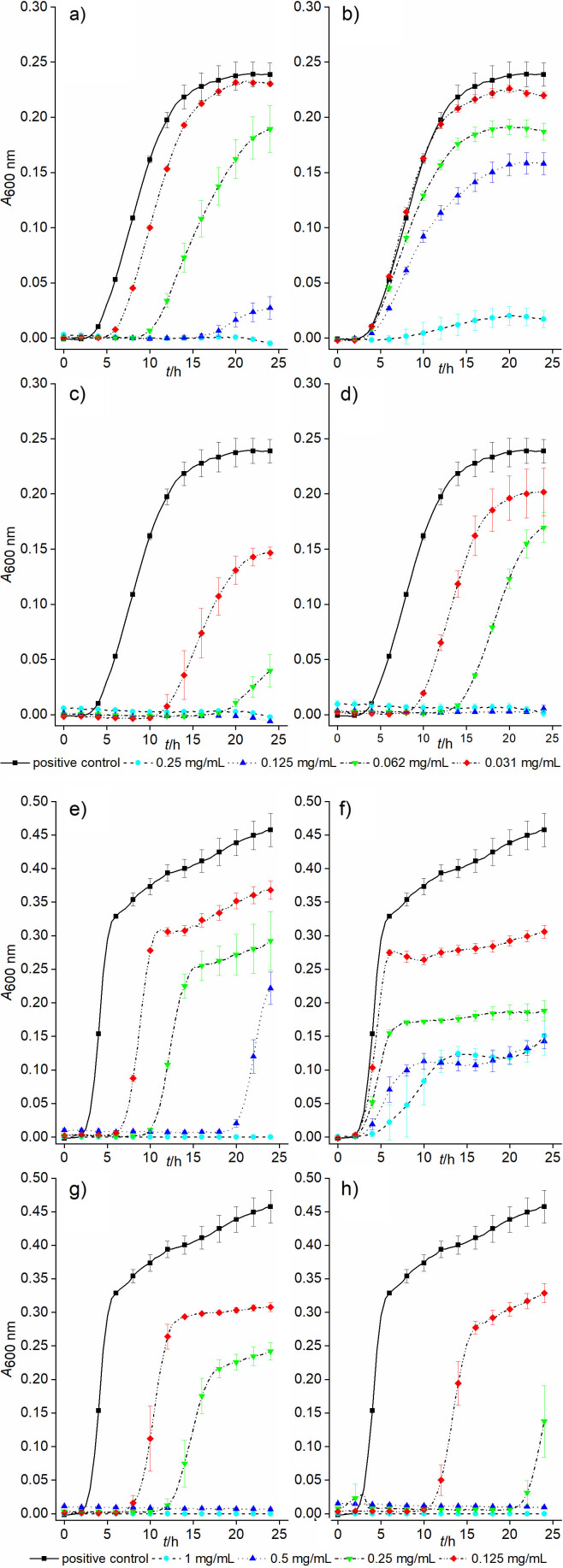
Effects of pure compounds: a and e) BITC, b and f) BCN, c and g) essential oil and g and h) extract of *T. majus* at different concentrations on the growth of: a-d) *S. aureus* and e-h) *E. coli*. Cultures were aerobically incubated for 24 h at 37 °C. Negative controls were deducted from the obtained results. Average values of *A*_600 nm_±S.D. are shown. BITC=benzyl isothiocyanate, BCN=benzyl cyanide

To evaluate their effect on the bacterial growth, pure compounds (BITC and BCN), essential oil and extract from *T. majus* seeds were used. *S. aureus* was exposed to plant preparations at concentrations from 0.25 to 0.031 mg/mL for 24 h. As can be seen in Figs. 2a and 2b, BITC and BCN at the concentration of 0.25 mg/mL completely inhibited *S. aureus* growth, while the effect of the essential oil and extract was even more pronounced (Figs. 2c and 2d). BITC at the concentration of 1 mg/mL had a bactericidal effect on the growth of *E. coli*, while BCN had no bactericidal effect at this concentration (Figs. 2e and 2f). The essential oil and the extract had a twofold lower bactericidal concentration than the BITC (Figs. 2g and 2h), again indicating that the combination of these two pure components is crucial for the antibacterial properties.

**Fig. 3:**
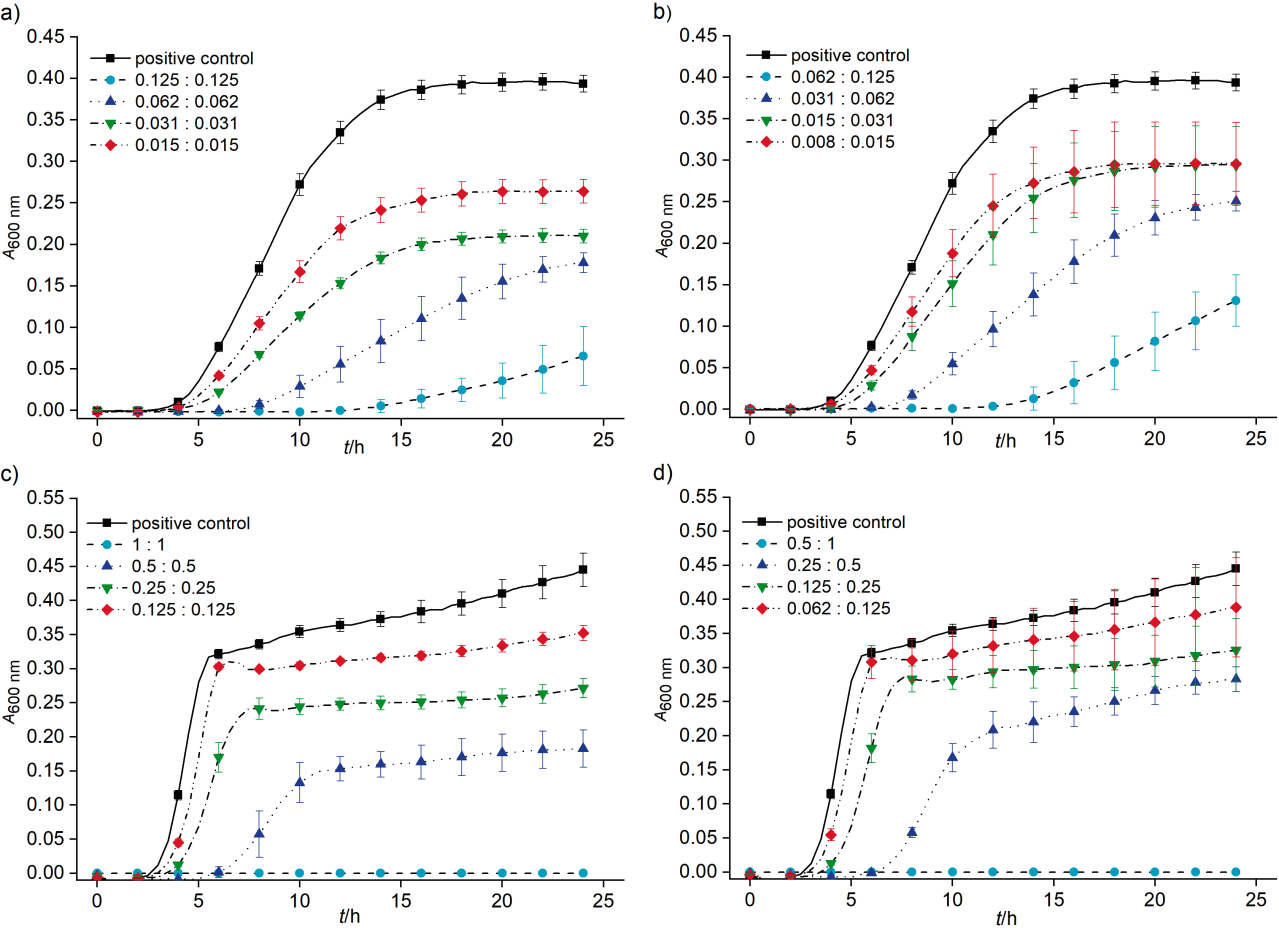
Effects of BITC and BCN at different ratios (1:1 and 1:2), determined regarding MIC values, on the growth of: a and b) *S. aureus* (MIC=0.125 mg/mL for BITC and BCN) and c and d) *E. coli* (MIC=0.5 and 1.0 mg/mL for BITC and BCN, respectively). Cultures were incubated aerobically for 24 h at 37 °C. Negative controls were deducted from the obtained results. Average values of *A*_600 nm_±S.D. are shown

Both the essential oil and the extract at the concentration of 0.125 mg/mL inhibited the growth of *S. aureus*. This stronger effect can be due to the combination of pure compounds BITC and BCN, which are an integral part of these preparations. To confirm this hypothesis, BITC and BCN were tested together at different ratios against *S. aureus.* When the ratio of BITC and BCN was 1:1, the effect was stronger than at the ratio 1:2 (Figs. 3a and 3b). When the concentration of BITC and BCN corresponded to their MICs, their effect was bactericidal, such as of essential oil and extract. When the concentration of BITC was half of that of BCN, there was no bactericidal effect, but inhibitory effect was observed. Even at lower concentrations both ratios of BITC and BCN (1:1 and 1:2) had inhibitory effects. These results indicate that BITC is the main compound that determines the antibacterial properties. This can also be noticed by observation of *S. aureus* growth curves when exposed to the essential oil, where even lower concentrations inhibited its growth. The inhibitory effect of the extract on the growth of *S. aureus* was weaker than of the essential oil, which can be explained by lower amount of BITC in the extract.

In order to obtain the same effect of the plant preparations on the growth of *E. coli* as on *S. aureus*, higher concentrations (ranging from 1 to 0.125 mg/mL) had to be used. It is known that Gram-negative bacteria are more resistant to treatments than Gram-positive bacteria, due to the composition of the outer membrane (*41*). Numerous studies have shown that disease resistance to a combination of compounds is less likely than to a single active component (*42*), which is also supported by our data on *S. aureus* and *E. coli*. Even lower concentrations of plant preparations had an inhibitory effect (Fig. 3b).

Similar to *S. aureus,* BITC and BCN at the ratios 1:1 and 1:2 showed that BITC is the dominant compound that induces higher antibacterial activity of the plant preparations. In the ratio corresponding to the obtained MICs, BITC and BCN had bactericidal activity (Figs. 3c and 3d). When the concentration of BITC was half of that of BCN, the inhibitory effect was weaker (Figs. 3c and 3d).

### Effect of volatile compounds from T. majus seeds on bacterial adhesion to polystyrene surface

**Fig. 4:**
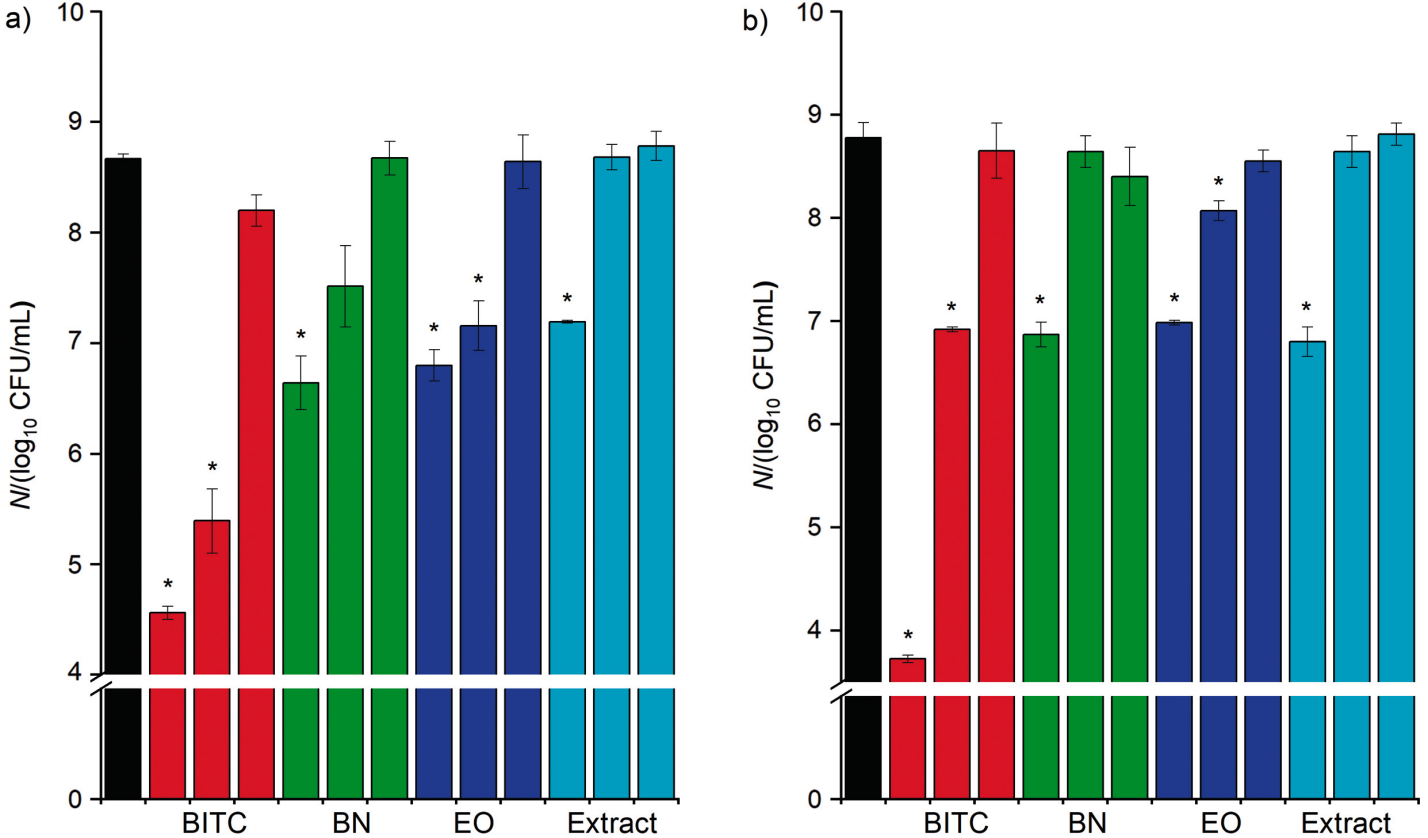
Effects of pure compounds (BITC and BCN), essential oil and extract of *T. majus* at MIC, ½ MIC and ¼ MIC on the adhesion of: a) *S. aureus* (MIC=0.125 mg/mL for BITC and BCN) and b) *E. coli* (MIC=0.5 and 1.0 mg/mL for BITC and BCN, respectively) to polystyrene surface. The results are expressed as mean value±S.D., *p<0.05. MIC=minimum inhibitory concentration, BITC=benzyl isothiocyanate, BCN=benzyl cyanide

Plant preparations obtained from the seeds of *T. majus*, *i.e*. essential oil and extract, and pure compounds BITC and BCN at MIC, ½ MIC and ¼ MIC were tested for their activity against the adhesion of *S. aureus* and *E. coli* to the polystyrene surface. It is important to use such low concentrations of plant preparations to affect not only bacterial growth, but also the properties important for bacterial phenotypes such as biofilm formation, virulence, quorum sensing and pathogenecity (*43*). Moreover, the use of such low concentrations may reduce the risk of developing bacterial resistance (*44*). ITCs are desirable compounds because of their ability to prevent biofilm formation and good efficacy on the established biofilms (*24*). As can be seen in Fig. 4, all preparations at MIC concentration significantly reduced the adhesion of *S. aureus* and *E. coli* to the polystyrene surface (p<0.05). BITC and essential oil significantly reduced the adhesion of *S. aureus* and *E. coli* to the polystyrene surface (p<0.05) also at half value of MIC. The lowest concentration did not have antiadhesive effect. The similar results for BITC and essential oil can be explained by the chemical composition of the oil, which consists mainly of BITC. Kaiser *et al*. (*24*) reported that even low ITC concentrations could inhibit biofilm formation and disturb biofilm viability, which is consistent with our results. BITC had the most pronounced antiadhesive effect against *S. aureus* and *E. coli*; it reduced the adhesion of *S. aureus* at MIC and the half value of MIC by 99.99 and 99.90%, corresponding to a reduction of 4 and 3 log CFU/mL, respectively. Similar observations were made against *E. coli*, where BITC at the MIC reduced the adhesion by 99.99%, which corresponded to a reduction of 4 log CFU/mL. All other preparations at MIC reduced the adhesion of both bacteria for more than 90%, which is more than 1 log CFU/mL. This is in line with the recommendations suggested by the European Food Safety Authority (*45*). The obtained results show that BITC is a promising natural compound with strong activity against the adhesion of *S. aureus* and *E. coli* to the polystyrene surface.

## CONCLUSIONS

Two volatile compounds were found in the essential oil and extract of *Tropaeolum majus* (garden nasturtium): benzyl isothiocyanate (BITC) and benzyl cyanide (BCN). The essential oil and extract rich in BITC showed highly active and moderately active, respectively, antiproliferative activity against three cancer cell lines: HeLa, HCT116 and U2OS, as well as high antimicrobial activity against *Staphylococcus aureus* and *Escherichia coli*. BITC had much higher inhibitory effect on all tested cells than BCN. Studies on the biological activities of isothiocyanates such as antiproliferative and antimicrobial ones, are of great importance as they have the potential to target different types of carcinoma or to extend the shelf life of food as natural preservatives in the food industry. One of the goals for the future may be the encapsulation of the *T. majus* plant extract so that a higher amount of BITC is biologically accessible and available.
